# Are Changes in Personality Traits and Alcohol Use Associated? A Cohort Study Among Young Swiss Men

**DOI:** 10.3389/fpsyt.2020.591003

**Published:** 2020-12-23

**Authors:** Gerhard Gmel, Simon Marmet, Joseph Studer, Matthias Wicki

**Affiliations:** ^1^Addiction Medicine, Lausanne University Hospital, University of Lausanne, Lausanne, Switzerland; ^2^Alcohol and Research Unit, University of the West of England, Bristol, United Kingdom

**Keywords:** personality traits, alcohol use, latent change scores, cross-lagged effects, young men

## Abstract

**Objective:** It is well known that certain personality traits are associated with alcohol use. Because less is known about it, we wished to investigate whether changes in alcohol use were longitudinally associated with changes in personality and in which direction the influence or causation might flow.

**Methods:** Data came from the self-reported questionnaire answers of 5,125 young men at two time points during the Cohort study on Substance Use Risk Factors (C-SURF). Their average ages were 20.0 and 25.4 years old at the first and second wave assessments, respectively. Four personality traits were measured: (a) aggression–hostility; (b) sociability; (c) neuroticism–anxiety; and (d) sensation seeking. Alcohol use was measured by volume (drinks per week) and binge drinking (about 60+ grams per occasion). Cross-lagged panel models and two-wave latent change score models were used.

**Results:** Aggression–hostility, sensation seeking, and sociability were significantly and positively cross-sectionally associated with both alcohol use variables. Drinking volume and these three personality traits bidirectionally predicted each other. Binge drinking was bidirectionally associated with sensation-seeking only, whereas aggression–hostility and sociability only predicted binge drinking, but not *vice versa*. Changes in alcohol use were significantly positively associated with changes in aggression–hostility, sensation seeking, and sociability. Associations reached small Cohen's effect sizes for sociability and sensation seeking, but not for aggression–hostility. Associations with neuroticism–anxiety were mostly not significant.

**Conclusion:** The direction of effects confirmed findings from other studies, and the association between changes in personality and alcohol use support the idea that prevention programs should simultaneously target both.

## Introduction

It is well known that personality traits are associated with alcohol use. A recent systematic review (1) showed that binge drinking was cross-sectionally associated with higher impulsivity and sensation-seeking, higher extraversion, and lower conscientiousness (inversely related to impulsivity and sensation-seeking). Similarly, other reviews have found heavy alcohol use to be associated with low conscientiousness (2), impulsivity (3), low agreeableness (inversely related with aggression–hostility), and high neuroticism (4). In addition, personality traits have been longitudinally associated with alcohol use. For example, Turiano et al. (5) found that higher neuroticism and extraversion, together with lower conscientiousness and agreeableness, were associated with higher alcohol use in midlife over a 9-year period. This was longitudinally confirmed by Hakulinen et al. (6) for extraversion and conscientiousness, but not for neuroticism (which was only cross-sectionally associated). Because few studies have done so, the present paper—based on a 5-year study of men aged 20.0 years old on average at baseline and 25.4 years old at follow-up—investigates the association between changes in alcohol use and changes in personality.

There are two extremes in personality theory, the “essentialist” and the “radical contextual” perspective (7). From the essentialist viewpoint [e.g., (8)] it is argued that personality traits like temperaments are endogenous dispositions (with a substantial genetic component), independent of environmental influences. According to McCrea et al. (8) (a) longitudinal twin and adoption studies show little influences of parenting, (b) long-term test-retest studies even in elderly show high predictability from personality assessment made 30 years earlier, which casts doubt on changes due to major life events in marital status, occupation, family composition, or physical health, and there is (c) remarkable consistency in personality development across cultures with different religions and radical different historical forces (e.g., in time of war). Although there is development from childhood to adulthood, this is seen as a biological process of maturation (similar to brain development), which is more or less completed around the age of 30 years. Essentialist do not deny environmentally conditioned changes in personality-related expressions. However, these are characteristic adaptations shaped by biologically based rather immune personality traits. Those taking a more radical contextual perspective see the importance in life changes and role transitions for in personality development, which takes it expression in low test-retest correlations particularly in time of rapid physical, cognitive and social changes (9), i.e., in the phase from childhood to emerging adulthood. Changes in personality are seen to be unlikely only genetic, biologically based changes (10), which is supported by epigenetic research, where biological aspects (i.e., temperament) influence personality vie adaptive processes [i.e., character, (11)]. With age environments become more stable, particularly also because of self-selection into social environments which reinforce personality (10) or pose fewer demands to cope or adapt to environmental stressors (12). Most of the differences between perspectives seems to be related to what actually is seen as temperament, personality or character, and there seems to be agreement that the phenotypical expression related with personality can change. Test-retest correlations of rank-order stability are smallest from childhood to early adulthood, but continue to increase beyond the age of 30 and are highest between ages 50 and 70. They also decrease with the increase of the time interval between measurement points, supporting change (7, 11, 12). As argued by Caspi et al. (7), genetic factors substantially influence personality traits, and gene environment-interactions may have effects on the genotype. However, longitudinal research neither supported a strict “essentialist” nor “radical contextual” as the rank-order stability changed with age, and wasnot as high as essentialists would claim, but still is relatively high and too high to take a “radical contextual” view.

Although individual's different personality traits are seen as relatively stable over time (12), normative mean-level changes in personality traits can occur across a life course. For example, agreeableness and conscientiousness increase from adolescence to middle adulthood (13, 14), whereas extraversion may decline (15). On the contrary, emotional stability (inversely related to neuroticism) has been found to increase (13). Pusch et al. (16), however, found neuroticism to be stable in late adolescence but to increase in young adulthood. Besides normative changes, the TESSERA framework [Triggering situations, Expectancy, States/State expressions, and Reactions; (14)] posits long-term changes in personality traits over time through repeated short-term sequences of change. TESSERA contains a multitude of aspects, including triggering situations, expectancy, reflective processes (e.g., self-reflection, assimilation, life reflection) and associative processes (e.g., implicit learning, reinforcement learning, habit forming). Through factors like reinforcement and reward, alcohol use may reflect such repeated short-term sequences and thus eventually result in changes in personality traits.

Although cross-sectional associations between personality traits and alcohol use, and longitudinal changes in alcohol use related to personality traits, are well-established, few studies have examined whether changes in alcohol use are related to changes in personality, or whether alcohol use predicts subsequent changes in personality. As argued by Littlefield et al. (17, 18), correlated change may have both theoretical and clinical relevance. Concurrent changes may be an indicator of a substantial, underlying, stable change relevant to treatment, whereby the personality change may support change in alcohol use. If individuals changed their alcohol use, but their personality did not, this may be an indicator of future relapse and recurrence. If there is correlated change, but no directional change, from alcohol use to personality, or from personality to alcohol use, there may be third-variable explanations (e.g., normative changes or genetic predisposition). If alcohol use predicts changes in personality, treatment should focus on reducing alcohol use to avoid any deterioration of unhealthy personality profiles. If personality predicts changes in alcohol use, then targeting interventions and treatment on personality may be a promising approach. Linking the treatment of alcohol use problems to personality traits has been shown to have long-term effectiveness in different countries (19–21).

The existing studies on alcohol use, which predicted personality changes or correlated changes between alcohol use and personality, are difficult to summarize for at least three reasons. First, they did not all use the same personality model and thus looked at different, although related, dimensions of personality traits. Second, they looked at different age segments (adolescents, young adults, older people), where normative changes may have had different impacts on personality changes. Third, they looked at different numbers of traits, e.g., five traits or a single trait such as neuroticism or aggression. Studies looking at multiple traits often showed mixed findings, i.e., whereas trait A was significant in one study, but not trait B, the opposite was true in another study.

In a general population study, Allen et al. (22) analyzed changes in the Big Five personality traits (extraversion, emotional stability, agreeableness, conscientiousness, and openness). They used regression models to predict personality at follow-up, adjusted for baseline personality and using the independent variables of alcohol use at baseline and change in alcohol use between baseline and follow-up. Of the Big Five, only changes in neuroticism were significantly associated with changes in alcohol use, but even this personality trait had a less than small effect size. Using latent growth modeling of the Eysenck Personality Questionnaire–Revised, Littlefield et al. (18) found medium effect sizes for positive associations between changes in alcohol involvement and changes in neuroticism and impulsivity during the period from 18 to 35 years old. No effect was found for extraversion. Hakulinen and Jokela (23) used a meta-analysis of six studies with populations in their early 50s, and with a mean follow-up of 5.6 years, to investigate the Big Five traits. Models looking at alcohol use at baseline predicted personality at follow-up (adjusted for baseline personality), although the effects were generally very modest. Hakulinen and Jokela (23) confirmed Littlefield et al.'s (18) study of emotional stability (inversely related to neuroticism), but, in contrast, they also found that alcohol use was associated with increasing extraversion. Luchetti et al. (24) used latent difference score models to follow people in middle and older adulthood over 4 years. Changes in alcohol use correlated positively with changes in extraversion, confirming Hakulinen and Jokela (23), but, in contrast, changes in alcohol use correlated negatively with changes in neuroticism.

Findings seem to have been most consistent for positive correlations with impulsivity/sensation-seeking or negative correlations with conscientiousness. Hicks et al. (25) compared normative declines in negative emotionality (comparable with neuroticism) and behavioral disinhibition (associated with sensation-seeking/impulsivity) from late adolescence (17 years old) to emerging adulthood (24 years old). Individuals with alcohol use disorders (AUDs) had higher cross-sectional scores for both personality traits than did individuals with no AUD at baseline and follow-up. Individuals on a desisting course of AUD exhibited less negative emotionality and less behavioral disinhibition at follow-up than those with a persisting AUD, almost returning to the levels of individuals who had never had an AUD. The authors concluded that personality differences may be associated with differences in alcohol use and AUD, but that the course of AUDs might affect changes in personality. Ashenhurst et al. (26) reported similar findings using latent class growth analysis. They followed individuals from their transition from high school to college, through their college years, and to the transition out of college. Changes in binge drinking generally paralleled changes in impulsivity and sensation seeking during college years, commonly with normative decreases in impulsivity and sensation seeking. The only group with non-normative increases in sensation seeking and impulsivity during their transition out of college was the group with higher binge drinking during their college years. Kaiser et al. (27) found that sensation seeking and alcohol use among college students bidirectionally reinforced each other, creating a vicious cycle, confirming White et al.'s (28) findings in adolescence and Quinn et al.'s (29) findings for the transition from high school and throughout college years. Hakulinen and Jokela (23) confirmed these aforementioned findings of studies in younger populations also for a population in their 50s, showing that increases in alcohol use were related to decreasing conscientiousness. Regarding the direction of effects, Littlefield et al. (17) using latent difference score models, found that correlated changes between alcohol use and novelty-seeking were better explained by the cross-lagged effect of personality influencing later alcohol use, rather than *vice versa*.

The link between alcohol use and aggression has been well known for a long time (30), and low agreeableness (inversely related to aggression–hostility) has been cross-sectionally associated with higher alcohol use (4). However, few studies have looked at the longitudinal associations running from alcohol use to aggression–hostility or low agreeableness. Hakulinen and Jokela (23) found that increasing alcohol use was associated with decreasing agreeableness. Najman et al. (31) did not look at aggression as a personality dimension, but rather at the aggressive and delinquent behaviors in four Australian samples. They found that aggressive and delinquent behavior preceded the onset of heavy episodic drinking (HED). However, the onset of HED was also consistently strongly associated with subsequent aggression–delinquency, even after adjusting for concurrent and past aggression.

The present study looked at four personality traits: aggression–hostility, neuroticism–anxiety, sociability (similar to extraversion), and sensation seeking. Changes in them were compared with changes in the volume of drinking and binge drinking. In addition, cross-lags were estimated from personality to alcohol use and from alcohol use to personality. Given the literature, if any associations were to be found, we hypothesized that changes in sensation-seeking should have the strongest positive associations with changes in alcohol use. The literature's findings are mixed with regards to neuroticism and sociability, and scarce with regards to aggression–hostility, but, if any changes in alcohol use were to correlate with changes in personality, these correlations were hypothesized to be positive. Furthermore, we hypothesized that the cross-lags for personality predicting alcohol use would be stronger than the cross-lags for alcohol use predicting personality, as personality is assumed to be more stable.

## Methods

### Participants

Data come from the first (hereafter baseline) and third (hereafter follow-up) wave of the Cohort Study on Substance Use Risk Factors (C-SURF). To avoid an overly heavy response burden, some questionnaire modules were only filled out in some waves. Personality was deemed to be relatively stable and was therefore not measured in wave two. At around 19 years of age, virtually all young Swiss men must attend a mandatory recruitment procedure to determine their eligibility for military or civil defense service. During this procedure, conscripts were invited to participate in C-SURF, with the assurance that assessments would be done at home, via the internet, and independently from the military or civilian defense services. During the general introduction by the army, all conscripts were first informed that they could voluntary participate in an ongoing study. While waiting for different army assessments during the army procedures, conscripts were approached by trained staff of C-SURF. They were verbally informed about the aims and received an information sheet about the aims of the study. If willing to participate, they received an informed consent form where they, by ticking boxes and final signature, had to confirm, that (a) they have been informed about the study, (b) they have read the information sheet attentively, (c) they can quit the study at any time without indication of any reason for quitting, (d) they confirmed to be contacted within the next months, (e) they may be contacted for further waves, which again would be voluntary, (f) information is confidentially, and will in no case be transferred to the army or other external people, and (g) they will be reimbursed by vouchers for shopping. Participants were compensated with vouchers of CHF 30 (about US$ 30) for the baseline assessment and CHF 50 for follow-up assessment of the present study.

Participants who preferred could fill out the questionnaire with a paper and pen. C-SURF was approved by the Human Research Ethics Committee of the Canton of Vaud (Protocol No. 15/07), and fully complies with the Helsinki declaration adopted first in 1964 and its amendments.

The baseline assessment occurred between September 2010 and March 2012, and 5,987 (79.2%) of the 7,556 conscripts who provided their written informed consent to participate completed the questionnaire. The follow-up assessment occurred between April 2016 and March 2018, and 5,125 (85.6%) of those who completed the baseline assessment also completed the follow-up.

### Measures

#### Personality

Three personality traits were assessed using the validated French and German versions of the cross-cultural, shortened form of the Zuckerman–Kuhlman Personality Questionnaire (32), namely aggression–hostility, sociability, and neuroticism–anxiety. Each trait was measured using ten true-or-false statements. We used means if respondents answered at least eight items for each dimension. Sensation seeking was measured using the Brief Sensation Seeking Scale (33) with eight items on a five-point Likert scale (“strongly agree” to “strongly disagree”). Scale means were used, if at least six items were answered. Means were then up-scaled to the original metric (sums). We did not measure personality disorders as indicated in diagnostic systems (ICD or DSM).

#### Alcohol Use Measures

First, we used an extended quantity–frequency (QF) measure, which asked separately for the usual number of drinking days on weekends (Friday, Saturday, and Sunday) and on weekdays (Monday–Thursday) over the past 12 months. Quantities per drinking day on weekends and weekdays had the following answer categories: 12 drinks or more, 9–11 drinks, 7–8 drinks, 5–6 drinks, 3–4 drinks, and 1–2 drinks. Frequencies and quantities were combined to yield the number of drinks per week and a volume-of-drinking measure. When related to the C-SURF sample, this measure has been previously shown to provide better associations with other variables than a generic QF measure and a 7-day diary (34).

Second, binge drinking was measured, using the standard question from the Alcohol Use Disorders Identification Test, as the consumption of six drinks or more (~60+ grams of pure alcohol with a standard drink of 10 grams) on an occasion in the past 12 months (response options: never, less than monthly, monthly, weekly, almost daily, or daily). This corresponds to the better known US measure of 5+ drinks for men with 12 grams of pure ethanol for a standard drink. The binge measure was converted into numbers of binge drinking days per year.

### Statistical Analysis

Descriptive changes between baseline and follow-up were tested using paired *t*-tests. Descriptive sample correlations with observed variables were tested using Pearson's correlations. These were purely descriptive tests, which do have assumptions of Gaussian error terms. Given our large sample size tests for non-normality would almost always yield significant deviations from normality (35), leaving researchers with their intuition to decide how severely the normality assumption is violated (36). Personality variables were very symmetrical with no violation of skewness, but showed higher peaks (positive kurtosis) for some variables. Alcohol use variables were, as commonly found in the literature right-skewed. Due to the central limit theorem with large enough sample sizes (>40) violations of normality do not cause major problems when using parametric tests (35). Additionally, Gaussian models are remarkably robust to even dramatic violations of the normality assumption and better than wrongly specified more complex models such as binomial or count models (36), which has been shown particularly for alcohol use variables (37).

We used observed variables at baseline and follow-up mainly of descriptive purposes. Main models were based on differences between follow-up and baseline, which showed normal skewness parameters (between −0.2 and 0.2) even for the differences in alcohol use. Still, for the fourth moment, the kurtosis, for which some higher kurtosis values than expected under normality assumption were found. Violations of normality in general result in conservative testing, and are thus unproblematic for type 1 errors. This is particularly true for kurtosis violations, where power of tests decreases, resulting in conservative testing, when kurtosis values increase (38). Moreover, the main statistical model used was a latent change score model, more precisely, the cross-domain coupling model (39). Mplus version 8.1 software was used for the latent change score analysis using maximum likelihood estimations with robust standard errors to account for any skewness in the observed variables.

Latent change score models were estimated using a full information maximum likelihood (FIML) method, which enabled the inclusion of participants with missing values under the missing at random assumption. Missing values were excluded from descriptive analyses, and the corresponding sample sizes are indicated. There were generally few missing values.

**Figure 1:**
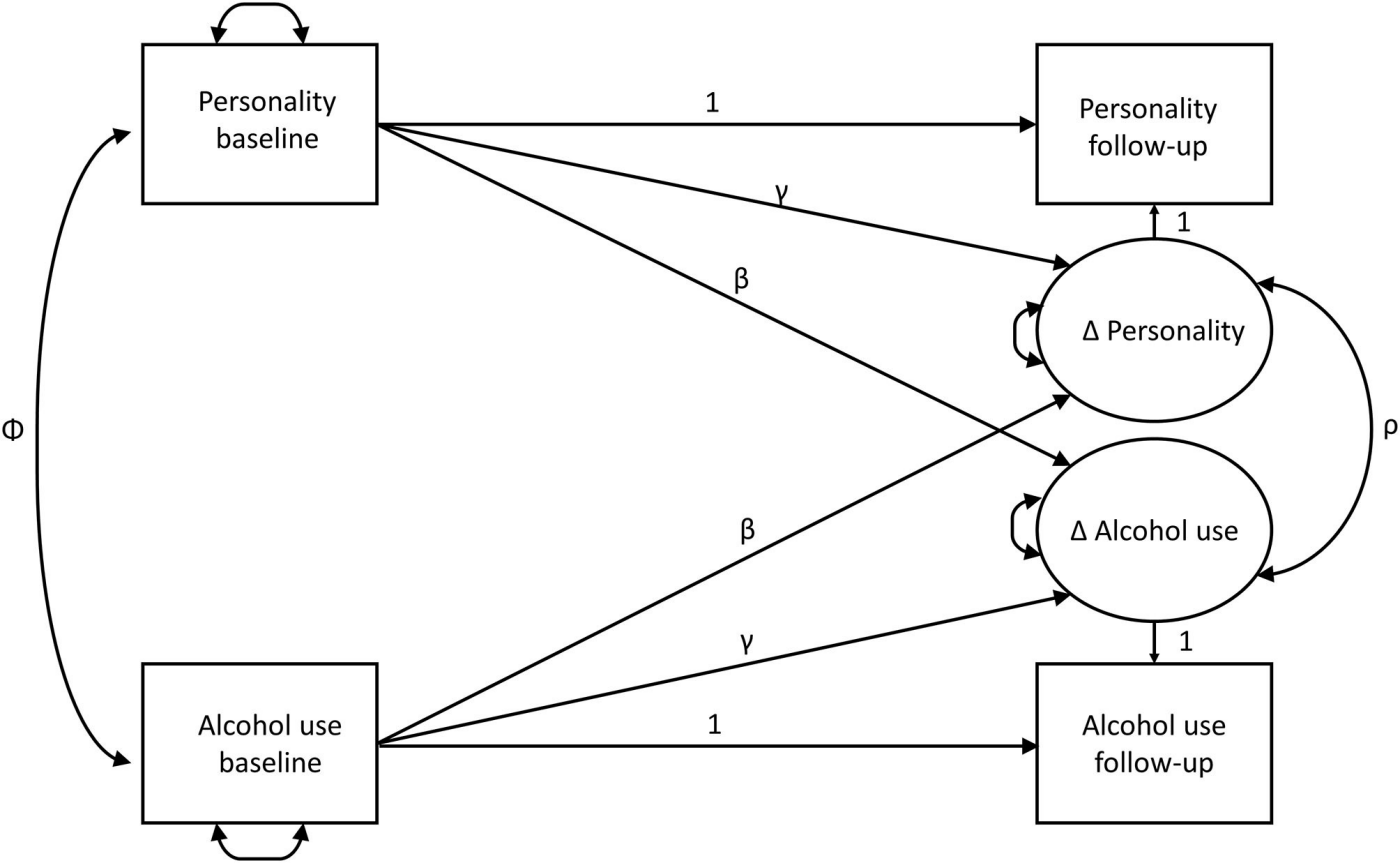
Theoretical latent change score model for alcohol use and personality traits. Latent variables are drawn as circles. Manifest or measured variables are shown as squares. Residuals and variances are drawn as double-headed arrows into an object. Correlations are drawn as double-headed arrows between two objects (Φ, ρ). Paths (β, γ) are drawn as single-headed arrows. Means are omitted for visual clarity.

Figure 1 shows the basic model which was estimated separately for each personality trait with binge drinking and volume of drinking. The Δs represent the latent change scores; the βs are the coupling parameters, which can be interpreted as cross-lagged coefficients, i.e., whether personality at baseline predicts change in alcohol use or alcohol use at baseline predicts changes in personality; and the γs are the paths from baseline measure to the corresponding change. This is important as these paths adjust for regression to the mean, which commonly occurs because individuals with high baseline measures tend to have lower follow-up measures on the same construct, and *vice versa*. This results in a negative association between the initial status and the change (40). Finally, ρ is the correlation between the latent change scores, reflecting the degree to which personality and alcohol changes co-occur, taking into account the coupling pathways (cross-lagged paths) and adjusted for regression to the mean.

**Figure 2:**
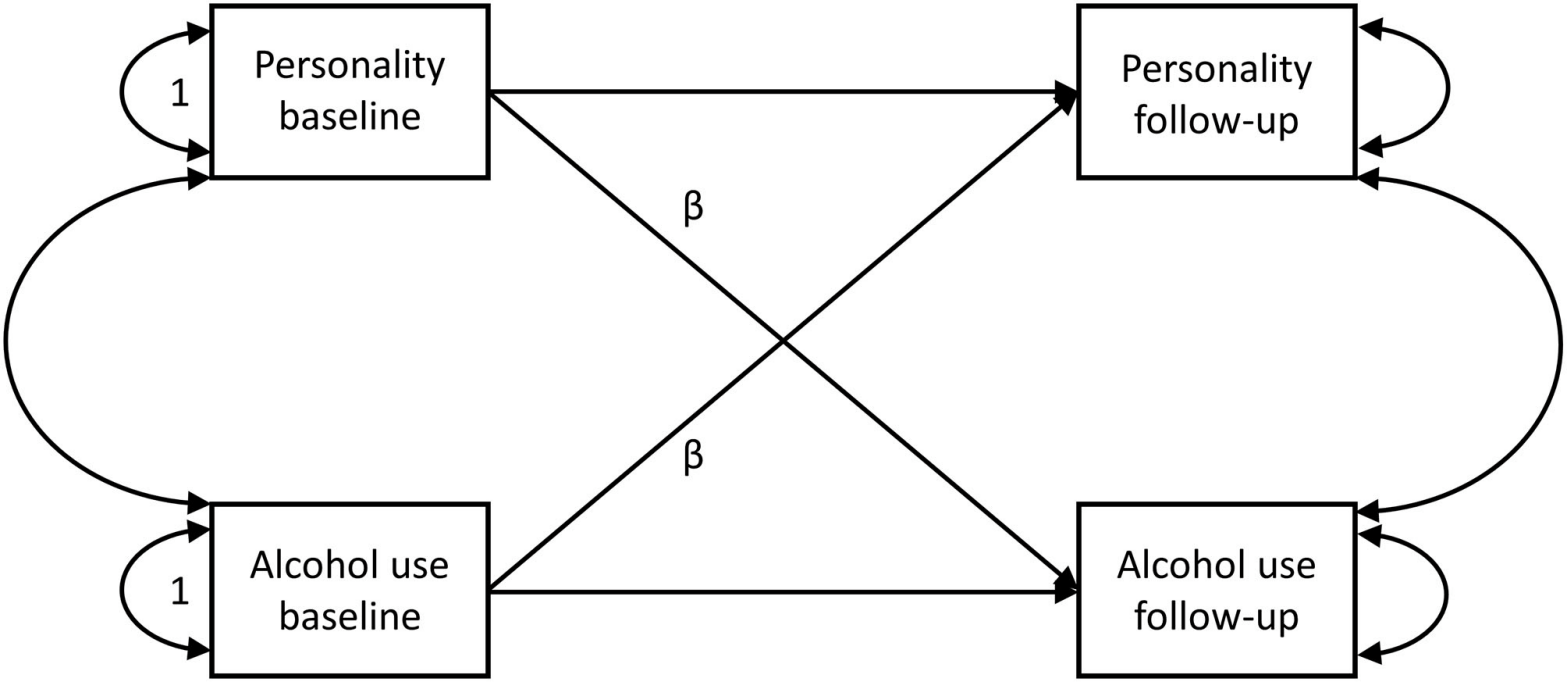
Theoretical cross-lagged model for observed variables of alcohol use and personality traits. βs are the cross-lagged paths.

Latent change score models have several advantages over models which analyze changes in observed variables, e.g., reducing measurement error. Additionally, as a sensitivity analysis, we calculated two further sets of correlations: firstly, between the changes in personality and alcohol use in observed variables (follow-up measure minus baseline measure); and secondly, we used the residuals from the two regressions (one for alcohol and one for each personality trait) of the observed change between follow-up and baseline measures on the baseline measures and then correlated the residuals to account for regression to the mean. We also used cross-lagged panel models (41) of the observed variables (see Figure 2) as a sensitivity analysis.

## Results

On average, participants were aged 20.0 (*SD* = 1.25) years old at baseline and 25.4 (*SD* = 1.22) years old at follow-up. The strongest cross-sectional correlations between personality and alcohol use were found for sensation seeking (Table 1). All the correlations were positive. Neuroticism showed the lowest correlation, which was even not significant at baseline. Correlations for aggression–hostility and sociability were in-between.

**Table 1:** Cross-sectional correlations between alcohol use variables and personality traits.

	**Baseline**			**Follow-up**		
	***n***	***r***	***p* value**	***n***	***r***	***p* value**
**Drinks per week (volume)**						
Aggression–hostility	5,085	0.181	<0.001	5,121	0.108	<0.001
Sociability	5,076	0.180	<0.001	5,117	0.155	<0.001
Neuroticism–anxiety	5,079	0.025	0.071	5,117	0.040	0.004
Sensation seeking	5,092	0.266	<0.001	5,121	0.241	<0.001
**Annual binge drinking occasions**						
Aggression–hostility	5,091	0.155	<0.001	5,116	0.086	<0.001
Sociability	5,082	0.158	<0.001	5,112	0.136	<0.001
Neuroticism–anxiety	5,084	0.023	0.103	5,112	0.031	0.028
Sensation seeking	5,097	0.247	<0.001	5,116	0.184	<0.001

*r is the Pearson correlation coefficient*.

Volume of drinking declined by about one drink per week and almost nine annual binge drinking occasions (Table 2). Given the large sample size, these changes were highly significant (*p* < 0.001). All personality traits showed significant, normative (*p* < 0.001) mean changes across the entire sample. Whereas, aggression–hostility, sociability, and sensation-seeking decreased, neuroticism–anxiety increased. There were strong negative correlations between baseline measures and changes in both alcohol use measures and personality measures, pointing to regression to the mean effects (Table 2). According to Cohen (42), effect sizes of correlation coefficients of 0.1 are small, of 0.3 are medium, and of 0.5 are large.

**Table 2:** Descriptive characteristics of changes in alcohol use and personality traits from baseline to follow-up.

	***N***	**Baseline**	**Follow-up**	**Difference Fu-Bl**	***t* value difference**	***p*-value of *t*-test**	**Correlation baseline- change**	***p*-value correlation**
**Alcohol use**								
Drinks per week (volume)	5099	8.16	7.13	−1.03	−7.42	<0.001	−0.571	<0.001
Annual binge drinking occasions	5099	39.03	30.25	−8.78	−9.39	<0.001	−0.621	<0.001
**Personality**								
Aggression–hostility	5,107	4.13	3.76	−0.38	−12.05	<0.001	−0.537	<0.001
Sociability	5,094	5.86	4.92	−0.94	−30.3	<0.001	−0.494	<0.001
Neuroticism–anxiety	5,097	1.96	2.18	0.22	7.52	<0.001	−0.454	<0.001
Sensation seeking	5,114	24.39	23.86	−0.53	−5.84	<0.001	−0.536	<0.001

*Fu-Bl is follow-up measures minus baseline measures*.

Table 3 presents correlations between changes in alcohol use and personality measures for (a) the latent change score model [*rho* (ρ) in Figure 1], (b) changes in the observed variables, and (c) between the residuals of the regression of observed change on observed baseline measures to adjust for regression to the mean. The patterns of these findings are consistent. All correlations were significantly positive (except for the correlation between neuroticism and binge drinking in the latent change score model; *p* = 0.051). Effects were larger for sociability (*rho* = 0.116, *p* < 0.001 in the latent change score model for volume) and sensation seeking (*rho* = 0.166, *p* < 0.001 for volume), reaching small effect size thresholds as per Cohen (42), but they were below this threshold for aggression–hostility (*rho* = 0.041, *p* = 0.007, for volume) and neuroticism–anxiety (*rho* = 0.044, *p* = 0.011, for volume).

**Table 3:** Correlations between observed variables and between latent change scores.

	**Latent change score model (*****n*** **=** 5,125)^*^				**Observed changes**					
		**95% Confidence Interval**				**Unadjusted**		**Regression to the mean adjusted**	
	**Standardized estimate**	**Lower Limit**	**Upper Limit**	***p*-value**	***n***	***r***	***p*-value**	***r***	***p-*value**
**Volume**									
Aggression–hostility	0.041	0.011	0.071	0.007	5,081	0.044	0.002	0.043	0.002
Sociability	0.116	0.086	0.146	<0.001	5,069	0.106	<0.001	0.113	<0.001
Neuroticism–anxiety	0.044	0.010	0.077	0.011	5,071	0.039	0.005	0.047	0.001
Sensation seeking	0.166	0.138	0.194	<0.001	5,042	0.141	<0.001	0.164	<0.001
**Binge**									
Aggression–hostility	0.050	0.021	0.080	0.001	5,082	0.068	<0.001	0.048	0.001
Sociability	0.107	0.078	0.137	<0.001	5,068	0.102	<0.001	0.095	<0.001
Neuroticism–anxiety	0.031	0.000	0.062	0.051	5,071	0.029	0.040	0.032	0.022
Sensation seeking	0.119	0.088	0.149	<0.001	5,043	0.105	<0.001	0.112	<0.001

* *The latent change score model used Full Information Maximum Likelihood (FIML) estimation, taking participants with missing values into account under the “missing at random” assumption*.

Table 4 shows the latent change score model's standardized cross-lagged (coupling) path coefficients (βs) (see Figure 1). For comparison, the standard cross-lagged model's results for observed variables only (Figure 2) are also presented in Table 4, but the results reported refer to the latent change score model. Generally, the coefficients from both models were very similar.

**Table 4:** Standardized path coefficients of cross-lagged (full coupling) models of volume and binge drinking with observed and latent change score variables (left) and only observed variables (right).

	**Latent change score full coupling**				**Observed variables cross-lagged panel**			
		**95% CI**				**95% CI**		
**Baseline -> follow-up**	**Standard estimate**	**Lower limit**	**Upper limit**	***p-*value**	**Standard estimate**	**Lower 95% CI**	**Upper 95% CI**	***p*-value**
**Volume**								
Aggression -> volume	0.044	0.020	0.068	<0.001	0.047	0.022	0.073	<0.001
Volume -> aggression	0.032	0.006	0.057	0.014	0.033	0.007	0.060	0.014
Sociability -> volume	0.021	−0.003	0.045	0.087	0.023	−0.004	0.049	0.091
Volume -> sociability	0.028	0.005	0.052	0.017	0.028	0.005	0.051	0.017
Neuroticism -> volume	0.003	−0.020	0.027	0.773	0.004	−0.021	0.029	0.773
Volume -> neuroticism	−0.002	−0.030	0.025	0.859	−0.002	−0.029	0.025	0.859
Sensation seeking -> volume	0.053	0.026	0.080	<0.001	0.057	0.028	0.086	<0.001
Volume -> sensation seeking	0.051	0.023	0.080	<0.001	0.052	0.023	0.081	<0.001
**Binge**								
Aggression -> binge	0.034	0.012	0.055	0.002	0.041	0.015	0.066	0.002
Binge -> aggression	0.001	−0.023	0.025	0.949	0.001	−0.024	0.026	0.949
Sociability -> binge	0.030	0.009	0.052	0.006	0.036	0.01	0.063	0.007
Binge -> sociability	0.008	−0.016	0.032	0.506	0.008	−0.016	0.032	0.506
Neuroticism -> binge	0.004	−0.019	0.026	0.744	0.004	−0.022	0.031	0.744
Binge -> neuroticism	−0.004	−0.029	0.018	0.785	−0.004	−0.029	0.022	0.785
Sensation seeking -> binge	0.049	0.025	0.072	<0.001	0.058	0.03	0.086	<0.001
Binge -> sensation seeking	0.053	0.028	0.074	<0.001	0.053	0.028	0.078	<0.001

*Both the cross-lagged panel model and the latent change score model used Full Information Maximum Likelihood (FIML), allowing for the inclusion of participants with missing values under the “missing at random” assumption. Neuroticism is the neuroticism–anxiety personality trait, and aggression is the aggression–hostility personality trait. CI is Confidence Interval*.

There were three main findings. First, neuroticism and alcohol use did not predict each other significantly, and this was true for volume of drinking and binge drinking. Second, with regard to the volume of drinking, cross-domain coupling (cross-lagged) effects, i.e., volume predicting personality and personality predicting volume, were of similar magnitudes (though not significant for the sociability to volume path). Third, as regards binge drinking, the effects of personality predicting changes in binge drinking were stronger than for binge drinking predicting changes in personality, except for sensation seeking. For example, the standardized path from aggression–hostility to binge drinking was β = 0.034 (*p* = 0.002), however, the path from binge drinking to subsequent aggression–hostility was β = 0.001 (*p* = 0.949). Bidirectional links of a similar magnitude could only be found for sensation seeking, i.e., for sensation-seeking to binge drinking β = 0.049 (*p* < 0.001) and from binge drinking to sensation-seeking β = 0.053 (*p* < 0.001).

## Discussion

The present study indicated that changes in alcohol use were associated with changes in personality, and it was therefore in line with other studies (18, 23, 24). As Littlefield et al. (17, 18) have argued, such associations may have theoretical and clinical relevance for treatment, relapse, and recurrence. If personality predicts changes in alcohol use, interventions targeting personality may result in substantial benefits, and if alcohol use predicts personality changes, then targeting drinking may help prevent detrimental changes in personality. This study found that volume of drinking in particular (though not necessarily binge drinking) had longitudinally reciprocal relationships with personality, which may create a vicious cycle (23, 27–29, 43).

Changes in all four personality traits were significantly associated with changes in both alcohol measures (except for binge drinking and neuroticism–anxiety, *p* = 0.051). The effects for aggression–hostility and neuroticism–anxiety were smaller than those for sociability and sensation seeking. As regards neuroticism–anxiety, not only were the correlations between changes very small, but all their transactional (cross-lagged) paths were also highly not significant. Indeed, these findings on neuroticism–anxiety were not unexpected. Although neuroticism has been found to be cross-sectionally associated with substance use disorders (4, 44), studies looking at parallel changes have found mixed results (18, 22–24, 45). In addition, meta-analytical findings point to the possibility that overall associations between neuroticism and alcohol changes may be mainly due to results found with women (1, 43). The present study, however, consisted entirely of men, which may explain the mostly not significant findings with regards to neuroticism and why neuroticism also had the smallest cross-sectional associations.

More surprising were the small effects between alcohol use and aggression–hostility. The association between increases in alcohol use and decreases in agreeableness (which is inversely related to aggression) was the strongest association among all personality traits analyzed in Hakulinen and Jokela's (23) study. The link between alcohol use and aggression has been well known for a long time (30). Najman et al. (31) recently showed that aggression predicted binge drinking and that binge drinking predicted aggressive behaviors after adjusting for past aggression. The present study found a small but significant association between changes in alcohol use and changes in aggression–hostility. The directed cross-lagged effects were bidirectional for volume of drinking, although the path from aggression to volume of drinking was larger (standardized path = 0.044) than *vice versa* (standardized path = 0.032). Aggression predicted binge drinking, but the path from binge drinking to aggression was almost zero (standardized path = 0.001, *p* = 0.949). Overall, this points more to an explanation of aggression leading to alcohol involvement rather than the other way around. As argued by Jones et al. (46) in a meta-review of personality and aggression, disagreeable individuals may be less able to regulate the negative effects related to aggression, thus predisposing them to act out.

The strongest positive associations between alcohol use changes and personality changes found in the present study were for sociability (related to extraversion) and sensation seeking (related to impulsivity and inversely related to conscientiousness). Conscientiousness is a tendency to be self-controlled and disciplined, and therefore studies almost consistently show that increases in conscientiousness or decreases in sensation seeking are related with decreases in alcohol use (5, 18, 23, 26–29). In contrast to Littlefield et al. (17), who found stronger evidence that personality predicted alcohol use than *vice versa*, the present study found that sensation seeking showed bidirectional (transactional) paths, of similar strengths, from drinking to personality and from personality to alcohol use, and this for both binge drinking and volume of drinking. As argued by Hakulinen and Jokela (23), alcohol use has been associated with poorer goal-directed decision making. With impaired control, cognitive processes may be weakened and implicit impulsive processes may start to dominate (47), which is related to low conscientiousness or high sensation seeking.

Personality may also influence the experiences had with alcohol, e.g., being more sociable. Sensation seekers may attribute more positive experiences to their drinking (27). Hence, individuals looking for intense sensations may be pushed into seeking positive experiences; however, their positive consequences are often overestimated, whereas their potential negative consequences are underestimated (3). This, in turn, reinforces alcohol use, which was supported by the bidirectional associations found between sensation seeking and both alcohol use measures, and which is in accordance with the corresponsive principle (7).

Sociability at baseline predicted binge drinking at follow-up, but binge drinking at baseline did not predict sociability (extraversion) at follow-up, whereas the volume of drinking did—which mirrored the findings of Hakulinen and Jokela (23). This may mean that sociability has a greater association with social drinking than with problem drinking (23), although some social events may result in binge drinking. This hypothesis is supported by Luchetti et al. (24), who found an attenuated normative decrease in extraversion among people drinking light-to-moderately. These authors argued that alcohol consumers were more outgoing and socially engaged than abstainers, and thus had more sociable (extraverted) personalities. Alcohol use enhances mood in extraverted individuals and hence they receive greater rewards from drinking (48).

Some limitations in the present study should be considered. The sample consisted only of men, and differences between men and women and their associations between alcohol use and personality have been shown previously [e.g., (43)]. Neuroticism in particular may be more strongly associated with alcohol use among women. We also analyzed rather broad personality traits. For example, sensation seeking can be broken down into elements such as susceptibility to boredom, disinhibition, or experience seeking (33). Similarly, other aspects of impulsigenic traits, such as negative and positive urgency, reward sensitivity, or lack of premeditation (3) could also have been investigated. Further studies should also look at potential mediators, such as stable relationships or marriage [e.g., (18)]. The measurement of volume of drinking uses a 12-month recall period, which may result in recall bias, because participants may not be able to recall their alcohol use over such a long period. As stated by Dawson and Room (49), a past-year reference period is recommended if alcohol use is linked with consequences, because consequences may occur rather rarely. Short-term measurements of e.g., 1 week result in too many abstainers during this week and results in poorer associations with outcome measures (50), which has been shown for the present sample (34). In addition, our binge drinking measure uses “an occasion” as time frame. The current binge drinking definition of the National Institute on Alcohol Abuse and Alcoholism (51) specifies that the corresponding amount (about 60 grams ethanol or more) needs to be consumed within 2 h in order to bring blood alcohol concentration (BAC) of a typical male adult to 0.08 g/dL of ethanol. As we used the standard AUDIT question, and particularly as we want to use the AUDIT for other research questions, we did not adapt the original wording of the AUDIT for the 2-h criterion. Our analysis concerns young adulthood, which has been described as demographically dense, in that it involves more life-changing roles and identity decisions than any other period in the life course (9, 52). Also, brains may not have been fully matured at baseline, but more so at follow-up. Therefore, personality changes can be particularly expected during this phase, which may be lower at later ages (7, 8, 12) and therefore also less sensitive to changes in alcohol use.

In conclusion, in the present study, changes in alcohol use were related to changes in personality. Such changes in personality have been reported previously as being greatest between late adolescence and emerging adulthood (13), and given the bidirectional associations between personality and alcohol use, preventive efforts targeting both simultaneously might be promising. Indeed, interventions like the “PreVenture” program, which simultaneously target alcohol use and personality traits such as sensation seeking, anxiety, or negative thinking, have shown long-term effectiveness in several different countries (19–21).

## Data Availability Statement

The study questionnaires are available at www.c-surf.ch. The dataset analyzed during the current study, is available at ZENODO, doi: 10.5281/zenodo.4114100. Gmel G, Marmet S, Studer, J, Wicki M. Data for are changes in alcohol use and personality traits associated? A cohort Study among young Swiss men [Data set]. *Zenodo*. doi: 10.5281/zenodo.4114100. All data of the project, properly anonymized are available via C-SURF's homepage www.c-surf.ch upon detailed research request.

## Ethics Statement

The studies involving human participants were reviewed and approved by Human Research Ethics Committee of the Canton of Vaud (Protocol No. 15/07). The patients/participants provided their written informed consent to participate in this study.

## Author Contributions

GG analyzed and interpreted the data and drafted the manuscript. MW, SM, and JS revised the manuscript critically for important intellectual content, helped with the analysis plan and statistical analysis, have given final approval of the version to be published, and have agreed to be accountable for all aspects of the work in ensuring that questions related to the accuracy or integrity of any part of the work are appropriately investigated and resolved. All authors contributed to the article and approved the submitted version.

## Conflict of Interest

The authors declare that the research was conducted in the absence of any commercial or financial relationships that could be construed as a potential conflict of interest.
